# Randomised controlled trial of Interpersonal and Social Rhythm Therapy and group-based Cognitive Remediation versus Interpersonal and Social Rhythm Therapy alone for mood disorders: study protocol

**DOI:** 10.1186/s12888-022-03747-z

**Published:** 2022-02-14

**Authors:** Katie M. Douglas, Maree L. Inder, Marie T. Crowe, Jennifer Jordan, Dave Carlye, Cameron Lacey, Ben Beaglehole, Roger Mulder, Kate Eggleston, Katherine A. Donovan, Christopher M. A. Frampton, Christopher R. Bowie, Richard J. Porter

**Affiliations:** 1 Department of Psychological Medicine, University of Otago, Christchurch, New Zealand; 2 Specialist Mental Health Services, Canterbury District Health Board, Christchurch, New Zealand; 3 Māori Indigenous Health Institute, University of Otago, Christchurch, New Zealand; 4 Department of Psychology, Queen’s University, Kingston, ON Canada

**Keywords:** Bipolar Disorder, Major Depressive Disorder, Cognitive remediation, Psychotherapy, Randomised Controlled Trial, Cognitive impairment, Trial protocol

## Abstract

**Background:**

Individuals with mood disorders frequently experience cognitive impairment, which impacts on the long-term trajectory of the disorders, including being associated with persisting difficulties in occupational and psychosocial functioning, residual mood symptoms, and relapse. Current first-line treatments for mood disorders do little to improve cognitive function. Targeting cognition in clinical research is thus considered a priority. This protocol outlines a prospectively-registered randomised controlled trial (RCT) which examines the impact of adding group-based Cognitive Remediation (CR) to Interpersonal and Social Rhythm Therapy (IPSRT-CR) for individuals with mood disorders.

**Methods:**

This is a pragmatic, two-arm, single-blinded RCT comparing IPSRT-CR with IPSRT alone for adults (*n* = 100) with mood disorders (Major Depressive Disorder or Bipolar Disorder) with subjective cognitive difficulties, on discharge from Specialist Mental Health Services in Christchurch, New Zealand. Both treatment arms will receive a 12-month course of individual IPSRT (full dose = 24 sessions). At 6 months, randomisation to receive, or not, an 8-week group-based CR programme (Action-based Cognitive Remediation – New Zealand) will occur. The primary outcome will be change in Global Cognition between 6 and 12 months (treatment-end) in IPSRT-CR versus IPSRT alone. Secondary outcomes will be change in cognitive, functional, and mood outcomes at 6, 12, 18, and 24 months from baseline and exploratory outcomes include change in quality of life, medication adherence, rumination, and inflammatory markers between treatment arms. Outcome analyses will use an intention-to-treat approach. Sub-group analyses will assess the impact of baseline features on CR treatment response. Participants’ experiences of their mood disorder, including treatment, will be examined using qualitative analysis.

**Discussion:**

This will be the first RCT to combine group-based CR with an evidence-based psychotherapy for adults with mood disorders. The trial may provide valuable information regarding how we can help promote long-term recovery from mood disorders. Many issues have been considered in developing this protocol, including: recruitment of the spectrum of mood disorders, screening for cognitive impairment, dose and timing of the CR intervention, choice of comparator treatment, and choice of outcome measures.

**Trial registration:**

Australian and New Zealand Clinical Trials Registry, ACTRN12619001080112. Registered on 6 August 2019.

**Supplementary Information:**

The online version contains supplementary material available at 10.1186/s12888-022-03747-z.

## Background

The spectrum of mood disorders includes Major Depressive Disorder (MDD) and Bipolar Disorder (BD). Mood disorders are highly recurrent, long-term conditions, and are among the leading causes of disability worldwide [1, 2]. In New Zealand, high prevalence and disability associated with mood disorders applies to all ethnic groups and causes significant interference with functioning and suffering [3]. Rates of relapse in BD for those receiving routine care (medication alone) are 43% within 12 months, 57% within 2 years, and 77% within 4 years [4]. In Christchurch (New Zealand), where care consists mainly of medication and case management, 40% of patients admitted to a Specialist Mental Health Service (SMHS) with a mood disorder were readmitted into a SMHS within a year of discharge (data from Canterbury District Health Board SMHS). Reducing relapse and achieving longer duration between episodes is important because sustained periods of recovery of between 1 and 3 years are associated with progressively lower future relapse [4]. It is thus vital for treatment research in mood disorders to examine interventions that can be used alongside medication in order to promote long-term recovery.

### Targeting cognition in mood disorder treatment trials

Cognition has been identified as an important treatment target in mood disorders in attempting to improve functioning and reduce relapse rates [5]. A substantial portion of patients with mood disorders exhibit cognitive impairment [6, 7] across a range of domains (learning and memory, attention, executive function, psychomotor speed, social cognition) [8, 9]. Cognitive impairment often persists into recovery [10], and relates to problems in occupational and psychosocial functioning [10, 11]. Aspects of cognitive impairment have also been associated with increased risk of relapse [12].

Evidence suggests that current first-line treatments for mood disorders do little to improve cognitive function. Regarding first-line medications, a large-scale randomised trial (*n * = 1008) comparing effectiveness of three common antidepressant medications (escitalopram, sertraline, venlafaxine) on cognitive outcomes showed no effect of any medication [13]. In line with this, mega-analysis using patient level data in those with euthymic BD reported no consistent effect of commonly prescribed mood stabilising or antipsychotic medications on cognitive function [14]. A recent systematic review examining evidence-based psychological therapies in mood disorders concluded there was minimal evidence of pro-cognitive effects across therapies and across diagnoses (MDD or BD) [15]. An exception was metacognitive therapy, incorporating simple cognitive training techniques (Attention Training Technique), which has shown preliminary evidence of improving aspects of cognitive function [16]. Clearly, interventions targeting cognitive function in mood disorders are required to be able to improve cognitive and functional recovery.

Cognitive Remediation (CR) is an intervention involving a combination of repetitive cognitive training, strategy coaching, transfer of cognitive skills to daily life, and techniques to overcome cognitively-challenging tasks [17]. Systematic reviews examining effectiveness of CR interventions in BD have reported preliminary pro-cognitive effects [18] or inconclusive results [19, 20]. Two recent randomised controlled trials (RCTs) in euthymic BD reported improved aspects of cognitive function, particularly executive function, following CR (10–12 weeks in duration). Strawbridge et al. [21] reported additional improvement in psychosocial functioning at treatment-end and 3-month follow-up, while Ott et al. [22] showed improvement in subjective cognitive function at treatment-end only. In MDD, meta-analysis of CR reported short-term, moderate pro-cognitive effects, and small effects on daily functioning and mood symptoms, but none of these effects were durable [23]. In comparison with CR trials in BD, MDD trials have generally been smaller, shorter in CR duration, more variable in whether CR commences in the acute or remitted phase, and have often focused on short-term symptom reduction rather than longer-term functional outcomes. Therefore, larger RCTs of CR interventions in mood disorders, examining durability of multiple outcomes, are required.

### Combining cognitive interventions with evidence-based treatments to promote full recovery

An overarching aim of our research is to develop treatment packages which promote functional and symptomatic recovery for individuals with mood disorders. The current trial incorporates medication management, psychological therapy, and targeted CR approaches in order to achieve this. Research indicates that CR interventions which integrate principles of psychological therapy (e.g., role-play, goal setting) alongside a therapeutic relationship produce better retention rates, and improved self-reported cognitive and functional competence compared with traditional CR approaches [24]. We selected Interpersonal and Social Rhythm Therapy (IPSRT) as our long-term therapy to provide alongside CR. IPSRT is a psychological therapy developed for BD which focuses on stabilising circadian and social rhythms (e.g., sleep), as well as improving interpersonal functioning. Data suggests a link between disrupted sleep and cognitive [25] and functional impairment [26] in BD. Thus, improving sleep patterns alongside a CR intervention has a solid theoretical basis. Furthermore, IPSRT has been shown to be effective in reducing depression symptoms and reducing risk of relapse in BD [27, 28] and similar preliminary effects have been reported in MDD [29]. A treatment package including IPSRT and CR, therefore, has potential to improve several key aspects of recovery from mood disorders.

### Development of the current trial

We have recently completed a pragmatic RCT [30] which examined a novel combination of CR, IPSRT and medication management for people with mood disorders. Individuals (*n* = 68) recently discharged from SMHS in Christchurch, New Zealand, were recruited into the trial and randomised to receive a 12-month course of IPSRT either with (IPSRT-CR) or without CR (IPSRT alone). For those randomised to IPSRT-CR, CR was incorporated into the 50-min therapy sessions from an early stage of treatment and continued for 12 sessions. However, the addition of CR to IPSRT did not significantly improve the primary outcome, Global Cognition, more than IPSRT alone. On the other hand, significantly greater improvement in psychosocial functioning and longitudinal depression symptoms was evident in the IPSRT group compared with the IPSRT-CR over the treatment period. During the trial, therapists had concerns about integrating CR into IPSRT at such an early stage of treatment. At study intake, 43% of the sample were ‘in episode’, and thus, therapists often prioritised stabilisation of mood over CR. This was a factor contributing to the comparatively low dose of CR (average of 7 h) provided in our trial compared with other recent RCTs of CR in BD [21, 22]. We therefore propose a follow-on RCT which will once again aim to optimise psychological treatment to improve cognitive function, and thereby, to improve functioning and mood outcomes in individuals with mood disorders. However, the CR component will, in this trial, be conducted at a later stage of treatment and will be delivered separately from ongoing IPSRT. For the first 6 months of the 12-month intervention period, all individuals will receive the same intervention; individual IPSRT. At 6 months, individuals will be randomised to receive additional group-based CR (8 sessions over 8 weeks) or no additional treatment. Shifting to a group approach should allow for a higher and more consistent dose of CR to be delivered, and at a point when individuals should have a more stable mood.

An issue for psychological treatment trials is often translatability into clinical practice [31]. Conducting trials in patients with very thoroughly defined characteristics has advantages for determining effectiveness of treatments and understanding their mechanisms but has disadvantages in translation since in clinical practice it is necessary and more feasible to deliver treatments to broader groups of patients. As with our previous RCT described above, the current trial will thus recruit individuals across the spectrum of mood disorders (BD and MDD) at a particular point in their contact with services; that is, on discharge from SMHS.

### Factors associated with cognitive impairment and response to cognitive interventions

Factors influencing response to CR are important to identify in order to be able to determine how to tailor interventions to individuals’ cognitive and clinical profiles. This, in turn, should maximise treatment engagement and success. One of the more consistent predictors of CR response is baseline cognitive performance, with poorer performance associated with greater improvement from CR interventions [32]. Less is known about the impact of subjective cognitive difficulties on CR treatment response, but the finding that subjective cognitive difficulties relate to relapse in a large MDD sample receiving antidepressant treatments [33] indicates the importance of investigating this factor further in this context. Several measures of subjective cognitive difficulties, as well as factors likely to be associated with subjective cognitive difficulties (rumination, metacognition, mood severity) are included in the current protocol to determine their impact on CR response in secondary and exploratory analyses. Other factors to be included in exploratory analyses include: childhood trauma, personality traits, circadian rhythm disturbance, and medication adherence (see Methods).

Measurement of key biological processes implicated in mood disorder aetiology and cognitive impairment will occur in this trial, including assessing levels of inflammatory markers (pro-inflammatory cytokines and C-Reactive Protein; CRP) [34–36], vitamin C [37, 38], and androgens (females only) [39]. To be able to comprehensively treat cognitive impairment, the underpinnings of cognitive impairment must be more fully understood; obtaining these biological data will help inform this understanding.

### The patient perspective

One important perspective that has been overlooked in most clinical trials of CR to date is the patient perspective of cognitive impairment, and of undergoing cognitive interventions. Cognitive and functional symptoms of mood disorders have been described by patients as some of the most debilitating and concerning aspects of the disorders [40], and qualitative research has highlighted the broad impact cognitive impairment has on peoples’ lives and sense of self [41]. More in-depth understanding of patient perspectives will be examined in a qualitative study embedded within this trial.

## Methods and design

**Fig. 1:**
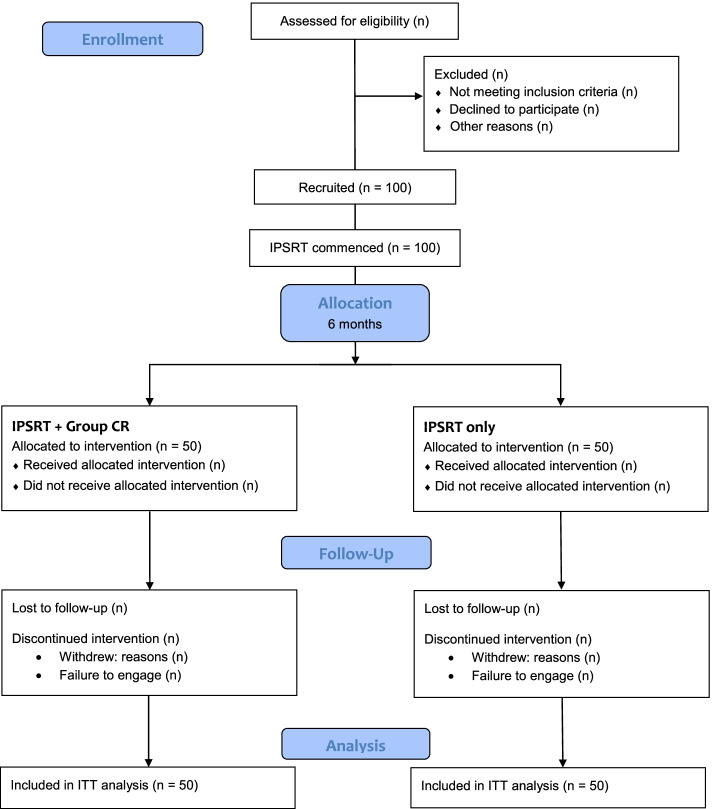
CONSORT flow diagram. The flow chart depicts participant progression through the study from initial enrolment through allocation, follow-up and analyses

See Fig. 1 for study flow and Table 1 for key time-points for interventions and assessments.

**Table 1:**
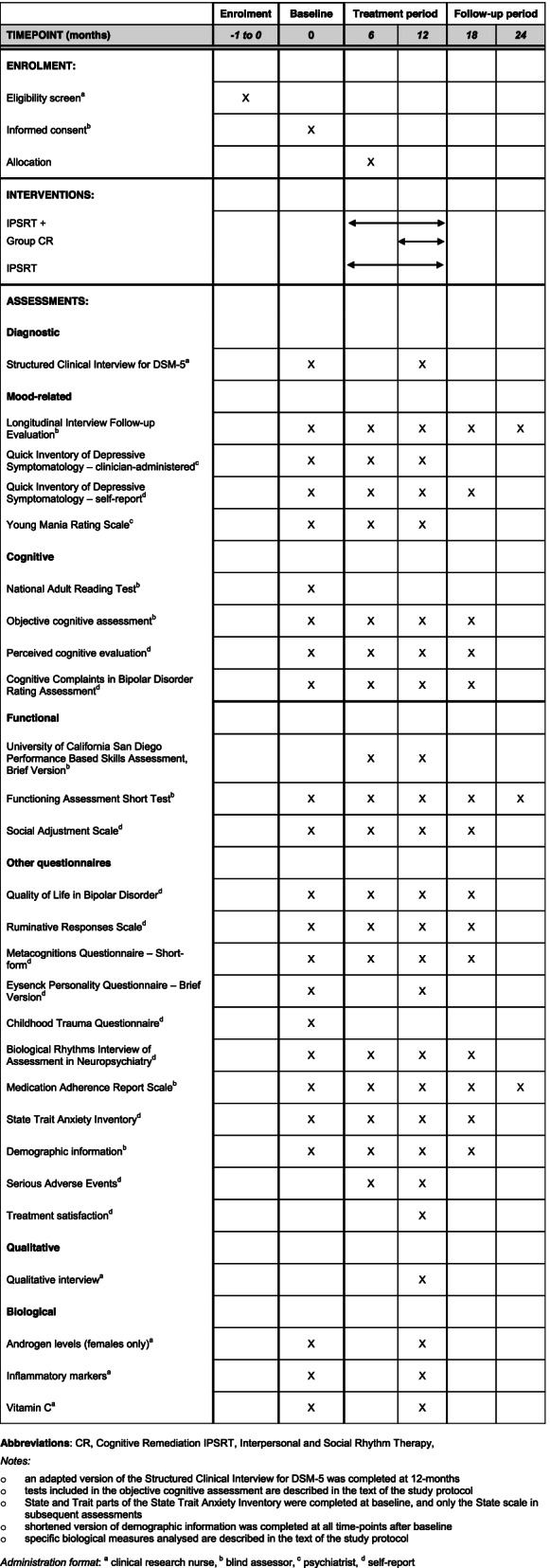
Schedule of enrolments, interventions, and assessments

### Aim

To compare the effectiveness of a 12-month intervention involving IPSRT combined with group-based CR (IPSRT-CR), with IPSRT alone, on cognitive functioning (primary outcome, Global Cognition), general functioning, and mood symptoms in individuals with mood disorders, at the point of discharge from SMHS in Christchurch.

### Primary hypothesis

Individuals randomised to receive IPSRT-CR will show significantly improved Global Cognition between 6-month (prior to commencing CR) and 12-month (treatment-end) time-points compared with individuals randomised to receive IPSRT alone.

Secondary hypotheses.

- Significant improvement in longer-term Global Cognition (18 months) will be found in IPSRT-CR versus IPSRT alone groups.
- General and psychosocial functioning will improve significantly more in IPSRT-CR versus IPSRT groups at treatment-end and follow-up time-points (18 and 24 months).
- Measures of mood symptoms (longitudinal depression symptoms, mood disturbance) will improve significantly more in the IPSRT-CR versus IPSRT alone groups at treatment-end and follow-up time-points.

### Trial design

See Fig. 1 for the study flow chart. This is a prospectively-registered (Australian and New Zealand Clinical Trials Registry; ref ACTRN12619001080112; 6 August 2019), two-arm, single-blinded RCT comparing IPSRT-CR with IPSRT alone for individuals with mood disorders. The trial was approved by the New Zealand Health and Disability Ethics Committees (Northern B) on 16 May 2019 (19/NTB/54). All participants will provide written informed consent. This RCT will be conducted and reported in accordance with SPIRIT (Standard Protocol Items; recommendation for Interventional Trials) [42] and CONSORT (Consolidated Standards of Reporting Trials) [43] guidelines. Recommendations specifically related to the design of CR trials from the International Society of Bipolar Disorder (ISBD) Targeting Cognition Taskforce have also guided development of this trial methodology [5].

### Setting

The study will take place in the Clinical Research Unit at the Department of Psychological Medicine, University of Otago, Christchurch, in collaboration with the Canterbury District Health Board.

### Participants

One hundred adults (≥ 18 years old) with MDD or BD (I, II, or Not Otherwise Specified) will be referred by treating clinicians at SMHS to the trial at the point at which individuals would typically have been discharged back to primary care. Diagnosis will be confirmed with the Structured Clinical Interview for DSM-5 Disorders, Research Version (SCID-5-RV) [44]. Patients must be identified by their referring clinician as showing evidence of cognitive impairment or identify this themselves. The screening question will be: “are you [or is the patient] having any difficulties with concentration, memory or decision-making?” (see Discussion for rationale). Exclusion criteria will include: schizophrenia or schizoaffective disorder, severe alcohol or drug dependence, history of severe brain injury (loss of consciousness greater than 1 h), previous course of CR or electroconvulsive therapy in past 12 months, serious medical or neurological condition affecting cognitive function, and pregnancy.

### Randomisation

Randomisation to the group-based CR intervention, or not, will occur at 6 months. This time-point has been chosen to avoid differential early dropout based on randomisation and to provide a period of mood stabilising treatment prior to CR treatment. Computerised permuted block randomisation will be undertaken by the team’s biostatistician (CF) prior to the commencement of the study and will be stratified according to mood disorder type (BD or MDD). Sequentially numbered envelopes will be stored in a locked cabinet by an independent research coordinator and given to therapists after the 6-month research assessment. Each patient will be allocated a randomisation number which will not be re-allocated if the patient drops out of the study.

### Interventions

#### Interpersonal and Social Rhythm Therapy

IPSRT combines Interpersonal Psychotherapy with a focus on social rhythms or routines in a person’s life. IPSRT will be delivered in an individual format by 8 clinicians (2 clinical psychologists, 5 mental health nurses, 1 social worker) trained in the provision of IPSRT, according to a manualised protocol. This has been adapted from the original IPSRT manual [45] by experienced IPSRT therapists (MI, MC) for patients with MDD as well as BD [29].

IPSRT will be conducted over a period of 12 months (weekly for the first 10–12 weeks, fortnightly for four months, and then monthly). Therapy frequency may increase, however, according to the patient’s mental state. A total of 24 sessions will be considered a full therapy dose, and 18 sessions as treatment completion.

#### Group-based cognitive remediation

CR involves engaging in cognitively stimulating activities that can produce changes in brain function [46]. We have previously employed an individual format for CR in our trials [30, 47], however, in order to increase patient engagement, to improve generalisability to ‘real-world’ functioning, and to allow for both IPSRT and CR components of therapy to be provided in a more structured and comprehensive manner, we have moved to a group-based CR format. Group-based CR is a well-established format in schizophrenia and mood disorders [48].

Group-based CR will involve weekly 90-min sessions over 8 weeks (i.e., 8 sessions). The group-CR manual used in the current study has been adapted for New Zealand from the original Action-based Cognitive Remediation (ABCR) manual developed by Professor Christopher Bowie (Queen’s University, Canada); we have termed it ABCR-NZ. Adaptations involved changes in language to reflect New Zealand English or te reo Māori, and changes to some of the ‘real-life’ tasks (described below) to reflect situations more realistic in New Zealand. We modified the frequency (once weekly) and duration (90 min) of CR sessions from the ABCR programme described in Bowie et al. [24]. We also reduced the number of modules covered (3 modules from ‘Speed and Attention’, 2 modules from ‘Learning and Memory’, 2 modules from ‘Executive Functioning’ and 1 module from ‘Social Cognition’; 1 module per session). These changes were made to reduce participant time burden, in the knowledge that many participants would be in full-time employment and many would still be attending fortnightly IPSRT sessions.

Each ABCR-NZ session involves four main components, described in detail in Bowie et al. [24]:

- *Computerised cognitive training*: Computerised cognitive training techniques are used with an online program called Scientific Brain Training Pro (SBT-Pro; www.scientificbraintrainingpro.com). SBT-Pro exercises use drill and practice procedures for training cognitive abilities including attention, processing speed, visual memory, verbal memory, working memory, and executive functions. Each exercise has several levels of difficulty that automatically adjust according to patients’ performance. They are game-like in nature and provide immediate feedback. A fixed schedule of exercises will be used wherein one exercise is practiced each group-CR session. For each module, a further two exercises relating to the module theme (e.g., learning and memory) can be practiced outside of sessions. During group-CR sessions, participants will complete exercises independently on a study tablet for approximately 20 min, with a therapist present to assist if required. In addition to the in-session computer exercises, participants are encouraged to practice these exercises for 30 min each day between group sessions, with therapists able to provide online feedback.
- *Strategy monitoring*: when participants are practicing computerised exercises, therapists will encourage them to think about strategies they are using. Following this, patients share these strategies with group members and therapists may facilitate this discussion and document strategies on a whiteboard.
- *Simulated real-life role-plays and tasks*: this involves engagement in simulated ‘real-world’ tasks and role-plays, as described in Bowie et al. [24]. These tasks are in line with the theme of each session. For example, in a group-CR session from the ‘Learning and Memory’ module, a real-life task involves a hypothetical work orientation day in which every group member introduces themselves (using a blurb provided by the therapist) and is then required to use cognitive strategies to recall information about other group members and then to introduce them to the group. Transfer strategies used in ABCR-NZ are tangible, procedural, and realistic.
- *Transfer discussion and cognitive activation*: the final component of each ABCR-NZ session involves group discussion about how computerised exercises and real-world simulations relate to activities in everyday life, followed by identification of cognitively-stimulating activities to work on between sessions.

CR treatment will occur in the final 6 months of each patient’s treatment, and will be commenced between approximately months 6 to 9 depending on patient numbers (see Discussion for rationale).

There will be 3 to 5 patients in each group, and two therapists. The trial lead, KD (Clinical Psychologist), received in-person training in ABCR in June 2019 from Professor Christopher Bowie and his lab members, and KD has trained all ABCR-NZ group therapists (4 mental health nurses, 1 psychiatrist) for the current protocol.

With regards to fidelity of both IPSRT and group-CR interventions, therapy sessions will be audio-taped and 10% randomly selected and rated to ensure adherence to IPSRT and CR therapy protocols using checklists for key components of each therapy (as in Douglas et al. [30]). Therapists participate in fortnightly group supervision, led by therapists with extensive experience in training and delivery of IPSRT (MI) and CR (KD).

#### Medication management

Six consultant psychiatrists will provide medication management. Patients will be accepted into the trial on any medication regimen, with treating psychiatrists using clinical judgment and the Royal Australian and New Zealand College of Psychiatrists clinical practice guidelines for mood disorders to inform medication management decisions [49]. The psychiatrist will see each patient at study entry, and at 6 and 12 months, as well as when requested by the patient’s therapist or the patient.

### Procedure

Patients will enter the study within 3 months of discharge from SMHS. After study entry, individuals will be assigned a psychiatrist for ongoing medication management and a therapist who will deliver IPSRT over 12 months. Baseline assessment will occur within one week of study entry, and will involve SCID-5-RV [44] administered by a research nurse to confirm mood disorder diagnosis and comorbidities, and self-report questionnaires assessing subjective cognitive function (Cognitive Complaints in Bipolar Disorder Rating Assessment, COBRA [50]), psychosocial functioning (Social Adjustment Scale, SAS [51]), depression symptoms (Quick Inventory for Depressive Symptomatology – Self Report, QIDS-SR [52]) and other key factors described below. A research assistant, blinded to randomisation, will conduct objective cognitive assessment (described below) and assess verbal IQ (National Adult Reading Test, NART [53]) in person, as well as measures of longitudinal mood symptoms (Longitudinal Interview Follow-up Evaluation, LIFE [54]), general functioning (Functioning Assessment Short Test, FAST [55]), and medication adherence (Medication Adherence Rating Scale, MARS [56]) via telephone, within one week of baseline assessment. The psychiatrist will complete the clinician-administered versions of the QIDS (QIDS-C [52]) and Young Mania Rating Scale (YMRS [57]) and a clinical research nurse will take blood samples for biological measures.

At 6 months, patients will undergo a second objective cognitive and functional (University of California San Diego Performance Based Skills Assessment, Brief Version (UPSA-B) [58]) assessment (blind research assistant), a telephone interview (same measures as baseline), a psychiatrist review (same measures as baseline), and self-report questionnaires (described below). Immediately following this, patients will be randomised to the group-based CR intervention, or not. The CR intervention will be provided for an 8-week period, commenced between approximately 6 months and 9 months. At treatment-end (12 months) objective cognitive and functional assessment will be repeated, along with all research measures conducted at baseline. Qualitative interviews and treatment satisfaction questionnaires will be completed at 12-months only by a research nurse trained in qualitative methodology. The same measures conducted at the 6-month time-point will be conducted at 18-months (with the exception of the UPSA-B), and at 24 months, three measures will be conducted via telephone (LIFE, FAST, MARS). Schedule of enrolments, assessments and interventions is presented in Table 1 and a populated SPIRIT checklist is provided in Additional file 1.

### Primary outcome

The outcome measures for the trial align with ISBD Targeting Cognition Taskforce recommendations for CR trials in BD [5]. A single composite score (Global Cognition) representing overall cognitive performance at each cognitive assessment (baseline, 6 months, 12 months, 18 months) will be calculated. Change in Global Cognition from 6 months (before randomisation to group-CR) to treatment-end (12 months) will be the primary outcome measure.

Cognitive function will be assessed with a battery of cognitive tasks designed to assess verbal learning and memory, visuospatial learning and memory, sustained attention, working memory, executive functioning, psychomotor speed and social cognition. These cognitive domains are included based on recommendations from the ISBD Targeting Cognition Taskforce [5], as well as research showing these domains to be significantly impaired in acutely-unwell patients with mood disorders [6, 59] and to be sensitive to the effects of Cognitive Remediation interventions [60]. The cognitive assessment will include the following tests:

- Rey Auditory-Verbal Learning Task [61]
- Brief Visuospatial Memory Test – Revised [62]
- Digit Span Forwards and Backwards [63]
- Trail Making Test – Part A and B [64]
- Delis-Kaplan Executive Function System—Fluency Battery (Category Fluency, Category Switching) [64]
- Digit Symbol Coding [63]
- Continuous Performance Test [62]
- Stroop Task [64]
- Negative Affective Priming Task [65]
- Reading the Mind in the Eyes Test [66]
- Facial Expression Recognition Test [67]

#### Power

This study is powered on the cognitive outcomes demonstrated in our preliminary RCT of Metacognitive Therapy versus Cognitive Behavioural Therapy for outpatients with MDD [16]. Metacognitive Therapy includes a simple cognitive training intervention (Attention Training Technique). A positive effect size difference between Metacognitive Therapy and Cognitive Behavioural Therapy was found on measures of visuospatial learning and attention (0.6) [16]. To detect a similar effect size for Global Cognition as statistically significant (2-tailed α = 0.05), 45 patients per group would be necessary for 80% power. We aim to recruit 50 patients per group. All statistical tests will utilise a 2-tailed *p*-value of < 0.05 to indicate statistical significance.

#### Analysis of primary outcome

Global Cognition will be calculated by averaging *Z*-scores across each cognitive domain, as listed above. A change *z*-score from 6- to 12-month follow-up will then be calculated using the Global Cognition score from these time-points. Change *z*-scores on Global Cognition will be compared using general linear models, with an intention-to-treat (ITT) approach. Treatment arm (IPSRT-CR versus IPSRT) and stratum (MDD versus BD) will be fixed factors, as in Douglas et al. [30].

### Secondary outcomes

See Table 1 for a summary of study measures (primary, secondary, exploratory) at each time-point.

#### Cognitive functioning

##### Objective cognition

Change in Global Cognition over all study time-points (up to 18 months) to determine durability of potential cognitive changes related to receiving group-based CR, or not. In addition, more specific analysis of cognitive function will involve determining changes in individual cognitive domains. Cognitive domains will include: (i) verbal learning and memory, (ii) visuospatial learning and memory, (iii) working memory, (iv) executive function, (v) sustained attention, and (vi) social cognition.

##### Subjective cognition

Self-reported cognitive functioning will be assessed using the COBRA [50], which was developed specifically for use in mood disorders. In addition, a questionnaire developed by study investigators to assess perceived cognitive performance during objective cognitive assessment will be conducted immediately following each objective cognitive assessment. Both measures will be conducted at all time-points up to, and including, 18 months.

#### General functioning

##### Objective functioning

‘Real-world’ functional competence will be assessed using the UPSA-B [58] at 6 and 12 months. This test uses role plays of everyday tasks focusing on communication skills and financial skills. Previous reports suggest that this measure is intermediate to neurocognitive abilities and actual real-world behaviour in mood disorders.

##### Subjective functioning

The FAST [55] will be used to assess self-reported general functioning. This interviewer-administered instrument has been validated in BD, and is commonly used in CR trials in mood disorders [5]. The FAST will be administered at all time-points (including 24 months) in the current study by a blind research assistant. The SAS will be used as an additional measure of social functioning, given the emphasis of IPSRT on the interpersonal domain [51], and will be completed at all time-points up to, and including, 18 months.

#### Mood symptoms

The LIFE [54] will be used to determine occurrence of episodes and sub-syndromal symptoms of MDD, hypomania or mania over the past 6 months (via telephone) at all study time-points (including 24 months). The LIFE has been extensively used in BD literature, particularly to measure number of episodes, including sub-syndromal episodes, in follow-up studies. A mean weekly score is generated based on the duration and severity of symptoms over a period of 6 months. The clinician-administered QIDS-C and YMRS will be conducted at all time-points up to, and including, 12 months to assess changes in mood symptoms over the course of the intervention period.

### Exploratory outcomes

Change in the following measures between treatment arms will be assessed as exploratory outcomes (see Table 1 for time-points of administration).

- Quick Inventory for Depressive Symptomatology – Self Report, QIDS-SR [52], as a measure of self-reported depression symptoms.
- Quality of Life in Bipolar Disorder (QoL-BD) [68]), a validated measure of quality of life for those with BD.
- Biological Rhythms Interview of Assessment in Neuropsychiatry (BRIAN) [69] to assess biological and sleep rhythms.
- Ruminative Responses Scale from the Response Style Questionnaire (RRS) [70], a measure of participants’ tendency to ruminate.
- Metacognitions Questionnaire – Short-form (MCQ-30 [71]) to assess individuals’ beliefs about their thinking processes.
- Eysenck Personality Questionnaire – Brief Version (EPQ-BV) [72] will assess individuals’ level of neuroticism and extraversion.
- MARS [56] will assess medication adherence in an interviewer-administered format.
- Biological measures: change in levels of inflammatory markers (including C-reactive protein levels (CRP) and key cytokines), androgens (Free Testosterone, Total Testosterone, Free Androgen Index, Sex Hormone Binding Globulin, Luteinizing Hormone, Follicle Stimulating Hormone Prolactin – females only), and Vitamin C will be analysed from blood samples.

#### Analysis of secondary and exploratory outcomes

To assess the effect of IPSRT-CR versus IPSRT on secondary and exploratory outcomes, general linear models will be used. These models will use treatment arm (IPSRT-CR versus IPSRT) and stratum (MDD versus BD) as fixed factors. Analyses will adopt an ITT approach.

#### Completers analysis (primary and secondary outcomes)

A sensitivity analysis will be undertaken using a completers analysis. This will include only participants who complete at least 18 IPSRT sessions, an adequate dose of CR based on number of group sessions attended and amount of computerised practice between sessions, and the relevant treatment-end assessments.

#### Missing data (primary, secondary and exploratory outcomes)

In the instance of incomplete data at any time-point from measures of primary, secondary and exploratory outcomes, a change of zero will be assumed from the previous time-point.

### Sub-group analyses

Performance on measures at baseline (described above), as well as change in these measures over time, will be used to determine effects on CR treatment response. Additional measures not described above that will be included in sub-group analyses will be the Childhood Trauma Questionnaire (CTQ) [73], the State Trait Anxiety Inventory (STAI) [74], and a Demographic Questionnaire.

#### Analysis of sub-group effects

Sub-group analyses will use general linear models, and will include sub-group measures and the interaction between treatment arm and these measures. Interaction terms will be used to evaluate the differential effects of CR treatment dependent upon the level of the baseline and change features.

### Qualitative interview

A semi-structured qualitative interview will be conducted at treatment-end (12 months) by a trained research nurse. Interviews will focus on the patients’ experience of their mood disorder, particularly on their cognitive and everyday functioning. Patients will also be asked about their experience of treatment and how they feel it impacted on their mood disorder symptoms. Qualitative data will be analysed using thematic analysis, as per Crowe et al. [41].

### Treatment satisfaction

Two scales developed by study investigators will be used to assess satisfaction and acceptability of IPSRT and CR components of treatment at the 12-month time-point. These scales measure responses on Likert-type scales, as well as providing space for comments from participants.

### Adverse events

Serious adverse events will be assessed using a Serious Adverse Events Questionnaire (as per [75]) at 6 and 12 months.

### Data management

An independent Data Safety and Monitoring Committee has been set up and will conduct 6-monthly meetings facilitated by, and with data prepared by, a member of the research team (CF). The committee consists of an international expert in psychotherapy trials, a New Zealand expert in clinical trials of psychotherapies and pharmacotherapies, and a New Zealand biostatistician.

### Protocol modification

Any substantive modification to the study protocol, particularly those related to patient selection, treatment, data collection, and study outcomes will be discussed by the study group and submitted to the Health and Disability Ethics Committees (New Zealand) for consideration. Any approved modification will be recorded on the ANZCTR and noted in any papers submitted for publication.

## Discussion

Findings from this prospectively-registered RCT will determine whether running an 8-week group-based CR programme alongside a 12-month course of IPSRT can improve cognitive functioning, general functioning, and mood symptoms over and above IPSRT alone. This is the first known RCT to combine a group-based CR programme with an evidence-based psychological therapy in mood disorders, even though it is well-established in schizophrenia that the combination of CR with rehabilitative therapies benefits wider functioning [76]. The sample will be recruited at a specific point in their contact with mental health services (on discharge) and will span the spectrum of mood disorders, allowing for greater opportunity to translate interventions back to clinical practice. Research measures are comprehensive in assessing objective and subjective aspects of key outcomes during the intervention and at longer-term follow-up points, and qualitative interviews will ensure that individuals’ perspectives of their mood disorder and its treatment are included. Primary and secondary outcomes are in line with ISBD Targeting Cognition Taskforce guidelines [5]. In designing the protocol for this RCT, various issues have required consideration, as follows.

### Spectrum of mood disorders

Individuals with MDD or BD will be recruited into the trial to be able to produce a more generalisable sample of relevance to community mental health services in New Zealand. We note, however, that most CR trials in mood disorders to date have recruited either BD or MDD samples. It is possible that having a mixed diagnostic sample may dilute any findings specific to BD or MDD. Indeed, in our previous RCT we found preliminary evidence of BD and MDD groups responding differentially to CR [30]. We plan, therefore, to include diagnosis type (MDD or BD) as a covariate in our analyses, to stratify randomisation according to diagnosis, and to examine effects specific to BD or MDD in exploratory analyses. The ability to conduct head-to-head comparisons between MDD and BD groups with regards to CR treatment response is a strength of this trial. Furthermore, in previous trials the diagnosis has changed during the 12-month intervention period on the basis of careful observation of, for example, mixed states and emergent or previously unrecognised hypomania. Including the spectrum of mood disorders therefore allows for a more considered examination of this spectrum.

### Screening for cognitive impairment

Due to our focus on developing interventions that can be translated back to clinical practice, it was important to have a screening protocol that could also be used in community mental health services. This was a key factor in choosing a subjective, rather than objective, screen for cognitive impairment. Further reasons were: (1) enhancing cognitive function can be beneficial for cognitive and functional outcomes even for those without objective cognitive scores below particular norm values [21] as there may still be change from previous level of functioning, and (2) individuals who self-report cognitive difficulties (subjective impairment) may be more motivated to participate in an intervention trial aimed to enhance cognition. Further, the ISBD Taskforce Targeting Cognition recommends screening for both objective and subjective cognitive impairment in clinical trials of cognitive interventions [5], and we agree that this is the most comprehensive approach for enriching CR trials. There is debate around the exact definition of objective cognitive impairment to use, and how potential decline from premorbid cognitive ability can be factored into this definition [6]. We therefore intend to explore the relation between objective and subjective cognitive impairment and treatment outcome in exploratory analyses of data.

### Choice of comparator treatment

This protocol involves an active comparison intervention (IPSRT), which has been shown to positively impact on mood symptoms and functioning [28, 29] and more preliminarily so, to aspects of cognitive function [77]. Utilising a non-active control intervention that does not produce behaviour change associated with a cognitively-enriched environment (e.g., treatment-as-usual) may result in stronger pro-cognitive effects. We are mindful, however, of difficulties with recruitment when referring clinicians and patients are aware of the possibility of being randomised to a non-active control intervention.

### Timing of CR intervention

Consideration was given to when group-based CR should be commenced within the 12-month intervention period. Most trials in MDD commence CR when patients are acutely unwell (in a recent meta-analysis, 16/20 trials of CR recruited MDD individuals who were ‘in episode’ [23]) and many focus on improving short-term mood and cognitive outcomes [23, 78]. On the other hand, BD trials often recruit patients when euthymic, and include assessment of longer-term functional outcomes. In our previous RCT [30], individual CR was commenced within the first two months of the 12-month intervention period, when patients were often still in episode. This led to IPSRT therapists reporting a perceived need to prioritise stabilisation of mood with IPSRT ahead of delivering CR. The current protocol therefore includes CR in the second half of the 12-month IPSRT course, giving patients the opportunity to learn strategies to stabilise circadian rhythms, routines, and mood symptoms prior to commencing CR.

### Dose of CR intervention

Dose of CR delivered in our previous RCT (an average of 7 h in total) was lower than we had aimed for, primarily due to issues outlined above in relation to therapists’ focus on mood stabilisation and complexities of integrating IPSRT and CR strategies within an individual therapy session. To our knowledge, there are no published comparative ‘dosing’ studies of CR interventions in mood disorders [78], however, effective programmes for individuals with MDD have entailed a wide range of sessions, from 6 to 64 [79]. In order to maximise the dose of CR provided for the current protocol, and to ensure consistency in delivery across participants, we have moved to a group-based approach which involves 12 h of ‘in-session’ CR and regular computerised practice and cognitively activating activities between sessions.

### Timing of research assessments

The trial protocol is unique in that randomisation occurs at 6 months, when all participants have received 6 months of individual IPSRT therapy. To be able to assess the specific effect of CR on the primary outcome, over and above any potential effect that stabilisation of mood and circadian rhythms may have on cognition, it was important to include objective cognitive assessment at 6 months and at treatment-end (12-months). We acknowledge that to be able to maximise detection of pro-cognitive effects from CR, cognitive assessment should occur immediately following completion of a CR intervention. However, participants will be commencing, and hence completing, CR at different points within the 12-month intervention period based on group numbers and sometimes, individual circumstances. Balancing these practical considerations with the potential burden of multiple research assessments for the sample, many of whom will be in full-time employment, led to development of our current time-line for research assessments; that is, every 6 months from study entry.

## Supplementary Information

**Additional file 1.** Populated SPIRIT 2013 checklist.

## Data Availability

Not applicable.

## Abbreviations

ABCR: Action-based Cognitive Remediation
ABCR-NZ: Action-based Cognitive Remediation – New Zealand
BD: Bipolar Disorder
BRIAN: Biological Rhythms Interview of Assessment in Neuropsychiatry
COBRA: Cognitive Complaints in Bipolar Disorder Rating Assessment
CR: Cognitive Remediation
CRP: C-Reactive Protein
CTQ: Childhood Trauma Questionnaire
EPQ-BV: Eysenck Personality Questionnaire – Brief Version
FAST: Functioning Assessment Short Test
ITT: Intention-to-treat
LIFE: Longitudinal Interview Follow-up Evaluation
MARS: Medication Adherence Rating Scale
MCQ-30: Metacognitions Questionnaire – Short-form
MDD: Major Depressive Disorder
NART: National Adult Reading Test
QIDS-C: Quick Inventory of Depressive Symptomatology – Clinician-administered
QIDS-SR: Quick Inventory of Depressive Symptomatology – Self-report
QoL-BD: Quality of Life in Bipolar Disorder
RCT: Randomised Controlled Trial
RRS: Ruminative Responses Scale
SAS: Social Adjustment Scale
SCID-5-RV: Structured Clinical Interview for DSM-5 – Research Version
SMHS: Specialist Mental Health Service
STAI: State Trait Anxiety Inventory
UPSA-B: University of California San Diego Performance Based Skills Assessment, Brief Version
YMRS: Young Mania Rating Scale
